# The impact of C216T and hot spot mutations of the TERT promoter on the clinicopathologic characteristics and S100A10 expression in papillary thyroid carcinoma: a comparative study

**DOI:** 10.1186/s13000-025-01613-6

**Published:** 2025-02-11

**Authors:** Ping Li, Chuqiang Huang, Xiaoling Liu, Huihui Gui, Jian Li

**Affiliations:** 1 Department of Pathology, Peking University Shenzhen Hospital, 1120 Lianhua Road, Shenzhen, 518036 Guangdong Province China; 2 Department of Thyroid and Breast Surgery, Peking University Shenzhen Hospital, 1120 Lianhua Road, Shenzhen, 518036 Guangdong Province China; 3 Department of Pathology, Shenzhen Second People’s Hospital, 3002 Sungang Road, Shenzhen, 518037 Guangdong Province China; 4 State Key Laboratory of Chemical Oncogenomics, Peking University Shenzhen Graduate School, 2199 Lishui Road, Shenzhen, 518000 Guangdong Province China

**Keywords:** Papillary thyroid carcinoma, TERT, C216T mutation, Hot spot mutations, S100A10

## Abstract

**Objective:**

The C216T mutation in the TERT promoter (TERTp) is a rarely reported genetic alteration in papillary thyroid carcinoma (PTC). Its clinical significance remains unclear. This study aimed to compare the impact of the C216T and hot spot mutations (C228T and C250T) of TERTp on the clinicopathologic characteristics and the expression of S100A10, a member of the S100 protein family, in PTC.

**Methods:**

In this retrospective study, a cohort comprising 8 PTC cases with the C216T mutation, 12 cases with the hot spot mutations, and 120 cases with the wildtype genotype was established. The influence of TERTp mutations on the clinicopathologic profiles of PTC was assessed.

**Results:**

The C216T mutation was mutually exclusive with the hot spot mutations and its frequency (0.19%) fell between that of C228T (0.68%) and C250T (0.06%). Compared to PTC cases with the wildtype genotype, cases with C216T mutations did not exhibit significant differences in clinicopathologic characteristics and S100A10 expression levels. In contrast, the hot spot mutations were positively associated with extrathyroidal extension (*p* = 0.001), ATA recurrence risk (*p* < 0.001), AJCC staging (*p* < 0.001), and increased expression of S100A10 (*p* = 0.005). Furthermore, a significant correlation was found between S100A10 expression and extrathyroidal extension (*p* = 0.005), lymph node metastasis (*p* = 0.013), and ATA recurrence risk (*p* = 0.023).

**Conclusion:**

The C216T mutation did not induce the aggressiveness of PTC as the hot spot mutations did. Furthermore, the hot spot mutations were closely associated with the increased expression of S100A10. The latter may contribute to the pro-invasive effect of the hot spot mutations on PTC.

**Supplementary Information:**

The online version contains supplementary material available at 10.1186/s13000-025-01613-6.

## Introduction

Papillary thyroid carcinoma (PTC) is the most common endocrine malignancy [1]. Over the last two decades, there has been a worldwide rise in the prevalence of PTC, which can be partially attributed to the increased rate of early detection resulting from advancements in imaging technology [2, 3]. Noteworthily, PTCs demonstrate an obvious heterogeneity in clinical course. The majority of tumors show biologically indolent behavior, while a minority display remarkable aggressiveness [4, 5]. Therefore, the exploration of molecular targets for the therapeutic and prognostic stratification has always been a research focus on PTC [6, 7].

Telomerase reverse transcriptase (TERT) is the catalytic component of telomerase and plays a crucial role in maintaining the length of telomeres by reverse transcribing hexameric repeats onto chromosomal ends [8]. TERT gene is located on chromosome 5 and consists of 16 exons, as well as a promoter region that spans 330 base pairs. Two hot spot mutations, C228T and C250T, are located in the TERT promoter (TERTp) at positions 1,295,228 (C > T) and 1,295,250 (C > T), respectively [9]. They also represent nucleotide changes of − 124 C > T and − 146 C > T, respectively, when sequenced from the translation start site [10]. Notably, the two mutations have been found to be associated with the invasive characteristics of PTC, such as enlarged tumor size, increased extrathyroidal extension, and metastasis [11–14].

The C216T mutation was recently identified in PTC by two separate research groups from Korea [15, 16], who reported one and four cases of this mutation, respectively. The C216T mutation of TERTp was located at position 1,295,216 (C > T) and situated at − 112 C > T upstream of the translation start site. It is interesting that the C216T mutation was also been uncommonly detected in lung adenocarcinoma and esophageal squamous cell carcinoma [17, 18]. Owing to the limited number of reports on this mutation to date, its clinical significance remains to be elucidated.

On the other hand, the regulatory mechanism of the TERTp mutations on the downstream signals has not been fully illustrated either. Some researchers have discovered that the hot spot mutations of TERTp could trigger the aberrant expression of several cancer-related genes, such as cellular retinoic acid binding protein 2 (CRABP2), murine double minute 4 (MDM4), and myotubularin related protein 3 (MTMR3) [19, 20]. Those genes might synergistically participate in the pro-invasive effect of the hot spot mutations on PTCs. S100A10 belongs to the S100 family of calcium-binding proteins [21]. Under physiological conditions, it engages with annexin A2 (ANXA2) to form a heterotetramer complex of ANXA2-S100A10 that is anchored on the cell surface. This complex can simultaneously interact with plasminogen and its activators, including tissue plasminogen activator (tPA) and urokinase-type plasminogen activator (uPA). As a result, plasminogen is transformed into plasmin, which subsequently triggers the degradation of the extracellular matrix, activation of metalloproteinases, and stimulation of angiogenesis [22]. Elevated levels of S100A10 have been observed in thyroid carcinoma and several other malignancies, such as ovarian cancer, pancreatic cancer, and colorectal adenocarcinoma [23–26]. This elevation consistently correlates with increased aggressiveness and metastasis in these tumors. Nevertheless, the potential association between the expression levels of S100A10 and TERTp mutations in PTCs has yet to be clarified.

In the present study, we therefrom investigated the following two issues: (1) Assessing the influence of the C216T mutation on the clinicopathologic characteristics of PTCs by comparing it with the wildtype genotype and hot spot mutations of TERTp. (2) Investigating the potential correlation between the expression levels of S100A10 and TERTp mutations.

## Materials

### Collection of study patients

**Fig. 1:**
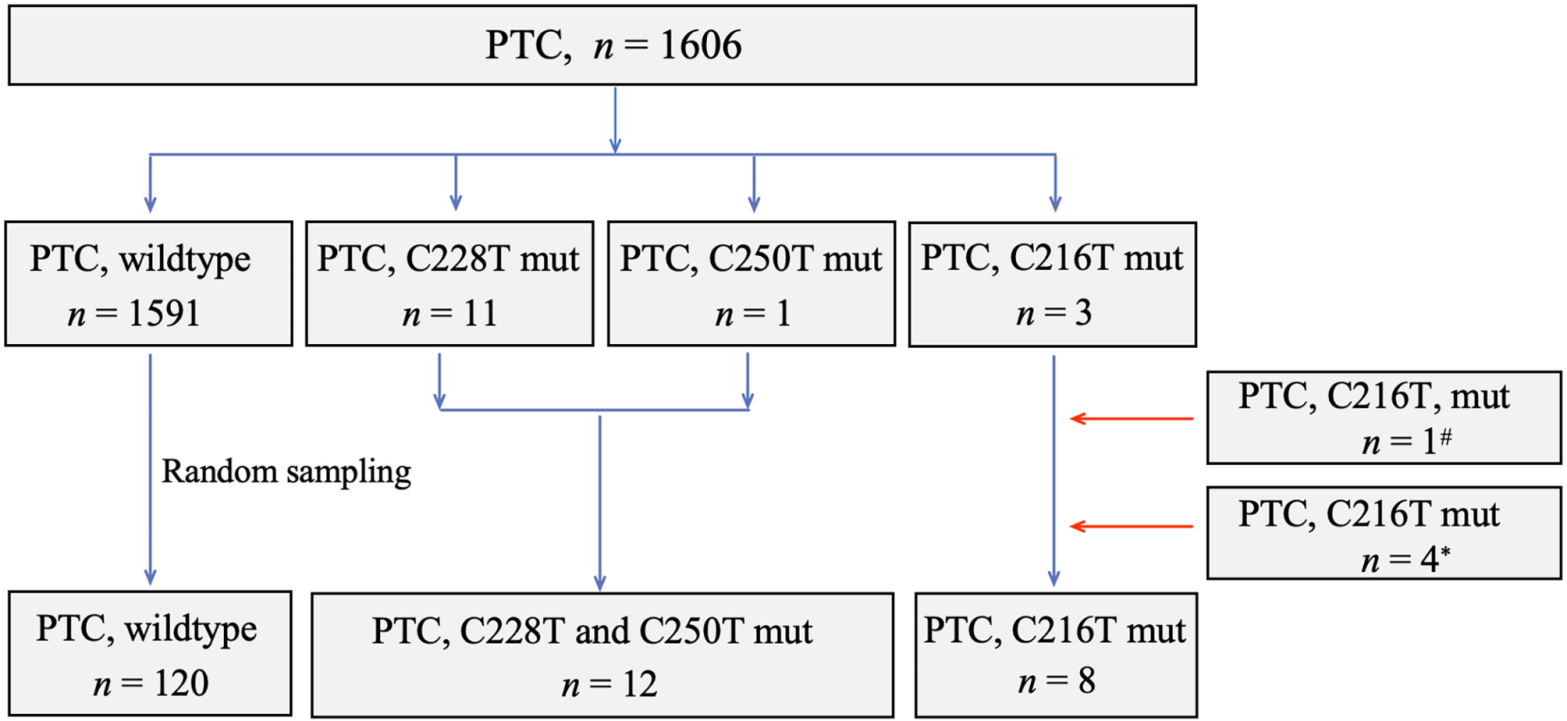
The flow chart outlining the process for establishing the study cohort PTC, papillary thyroid carcinoma; mut, mutation; # enrolled from reference 15; * enrolled from reference 16

Consecutive PTC patients, who underwent thyroidectomy and performed TERTp mutation assay at Peking University Shenzhen Hospital and Shenzhen Second People’s Hospital during the period of April 2021 through September 2023, were retrospectively retrieved for the study. A total of 1606 cases were identified during the retrieval process. Among these cases, 11 cases harbored the C228T mutation, 1 case harbored the C250T mutation, 3 cases harbored the C216T mutation, and 1591 cases exhibited wildtype genotype. Subsequently, the study cohort was established, comprising 15 cases with TERTp mutation and 120 wildtype cases. The latter was randomly collected from 1591 PTC patients with wildtype genotype. Moreover, 5 additional PTC cases with the C216T mutation, previously documented in literature [15, 16], were included in the cohort. As a result, our entire study cohort consisted of 8 PTC cases with C216T mutation, 12 cases with hot spot mutations (C228T and C250T), and 120 cases with wildtype genotype. The flowchart illustrating the process of subject collection was displayed in Fig. 1.

### Collection of clinicopathologic data

The demographic data of the enrolled subjects from our institution was obtained from the electronic medical records. For each case, the clinical and pathologic variables were re-evaluated and confirmed by clinician (X.L.L) and senior pathologists (H.H.G. and J.L.) according to the 8th edition of the American Joint Committee on Cancer (AJCC) staging system, the 2015 American Thyroid Association (ATA) guideline [27], and the 2017 WHO classification of endocrine organs tumors. Specifically, minimal extrathyroidal extension was defined as a microscopically detected invasion of perithyroidal tissues [28]. Gross extrathyroidal extension was defined as strap muscle invasion or invasion of the larynx, trachea, esophagus, recurrent laryngeal nerve, mediastinal vessels, carotidartery and/or prevertebral fascia [29, 30]. The diagnosis of Hashimoto’s thyroiditis was confirmed based on the following histopathological features: the presence of atrophied thyroid follicles and Hurtle cells, diffuse lymphoplasmacytic infiltration, and proliferated lymphatic follicles, with or without the interstitial fibrosis [31, 32]. The aggressive histologic variants of PTCs encompassed the tall cell and solid variants, while the non-aggressive variant included the classic, follicular, and diffuse sclerosing variants [33].

The clinicopathologic data of the 5 ciated cases with the C216T mutation was acquired from the relevant literature [15, 16]. As illustrated in the literature, all five cases were staged according to the 8th edition of the AJCC staging system. Four cases with available ATA risk stratification were adhered to the 2015 American Thyroid Association (ATA) guideline. Two cases labeled with minimal extrathyroidal extension were defined using the same criteria as our definition of minimal extrathyroidal extension outlined above.

### Assay for TERT promoter mutations

The PTC cases from our institution were tested for TERTp mutation by Sanger sequencing method, as stated below. Furthermore, the test was also conducted on the peritumoral normal thyroid tissues of the cases with mutant TERTp using the same method. Total DNA was extracted from 10 μm-thick sections of paraffin-embedded tissues using the QIAamp DNA FFPE Tissue Kit (Qiagen, Hilden, Germany) following the manufacturer’s protocol. The amplification product was 410 bp in length and contained the C216T, C228T, and C250T mutation sites. The primers used in the PCR reaction were forward 5’-ACATCATGGCCCCTCCCT-3’ and reverse 5’-CTGCCTGAAACTCGCGCC-3’. The reaction commenced with denaturation at 95 °C for 3 min, succeeded by 45 cycles of denaturation at 94 °C for 15 s, primer annealing at 60 °C for 45 s, extension at 60 °C for 45 s, and a final elongation at 70 °C for 5 min. The PCR products were finally sequenced on the 3500 Dx DNA analyzer (Applied Biosystmes, CA, USA).

### Assay for the BRAF V600E mutation

The PTC cases from our institution underwent testing for BRAF V600E mutation by the method of real-time fluorescence PCR. The DNA extraction procedure was consistent with that utilized in the TERTp mutation assay. The BRAF V600E Diagnostic Kit (AmoyDx, Fujian, China) was utilized for the detection. The amplification reaction volume was 50 μl, comprising Mix buffer, BRAF Taq polymerase, and DNA sample. The reactions proceeded according to the following protocol: 1 cycle at 95 °C for 5 min, 15 cycles at 95 °C for 25 s, 64 °C for 20 s, and 72 °C for 20 s, 31 cycles at 93 °C for 25 s, 60 °C for 35 s, and 72 °C for 20 s. The fluorescent signals were recorded at 60 °C.

### Immunohistochemistry

The PTC cases from our institution were immunostained with S100A10. Immunohistochemistry was performed utilizing a Ventana Benchmark^®^ XT autostainer (Ventana Medical Systems, AZ, USA). The slides were incubated with S100A10 primary antibody (1:200; ab76472, Abcam) for 24 min at 37 °C. The specimens were subsequently incubated with an ultraView Universal HRP multimer (Ventana) and visualized utilizing the ultraView Universal DAB Detection kit (Ventana).

### Evaluation of the immunostaining score of S100A10 protein

A semi-quantitative scoring method was employed to measure the immunohistochemical (IHC) score for S100A10, taking into account the staining intensity and the proportion of positively stained tumor cells. The staining of S100A10 was present in the cell membrane, with or without cytoplasm staining. Cancer cells that exhibited strong cell membrane or membrane and cytoplasmic staining were considered to have strong staining intensity (SSI). The benchmark for SSI was shown in Supplementary Fig. 1. The staining intensity in cancer cells that was weaker than that of SSI was categorized as weak staining intensity (WSI). The scores for WSI and SSI were assigned the values 1 and 2, respectively. The proportion of cancer cells (PC) classified as SSI or WSI was assigned a score of 1, 2, 3, 4, or 5 based on the percentage: < 10%, 10% to < 25% (one quarter), 25% to < 33.33% (one third), 33.33% to < 50% (one half), or ≥ 50%, respectively. The IHC score was determined using the formula (SSI score × corresponding PC score) + (WSI score × corresponding PC score).

### Analysis of the Cancer Genome Atlas (TCGA) data

The clinical information and mRNA-seq data of thyroid cancer, as well as the TERT mutation profile, were obtained from the TCGA database (https://portal.gdc.cancer.gov/) and Cancer Genome Atlas Research Network report (10.1016/j.cell.2014.09.050) [34], respectively, on 12 October 2022. The mRNA data were normalized using the IlluminaHiSeqRNA-seq V2 platform. Among the 507 thyroid cancer cases recorded in the TCGA database, 495 were definitely documented as PTC cases and contained intact S100A10 mRNA data. Out of the 495 cases, 26 cases exhibited the C228T mutation, 8 cases showed the C250T mutation, 1 case presented the C228A mutation, 342 cases displayed the wildtype genotype, and the remaining 118 cases lacked available information on the status of TERTp mutation.

### Statistical analysis

Statistical analyses were conducted using SPSS Ver.22.0 software (IBM Corporation, NY, USA). The association between TERTp mutation and the clinicopathologic data were analyzed using the Chi-square test or Fisher’s exact test for categorical variables. The correlation of continuous variables between TERTp mutation, S100A10 levels, and the clinicopathologic data were compared using Student’s t-test. A two-sided *p* < 0.05 was considered statistically significant.

## Results

### The frequency of TERTp mutations

Among the 1606 consecutive cases of PTC analyzed for TERTp mutations at our institution, 11 cases (0.68%) exhibited the C228T mutation, while 3 cases (0.19%) and 1 case (0.06%) were identified with the C216T and C250T mutations, respectively. The overall mutation rate was 0.93% (15/1606). Additionally, the three types of mutation were mutually exclusive. Furthermore, all the mutations observed were somatic, as the peritumoral normal thyroid tissues from the respective cases tested negative for these mutations.

### The baseline data of the study cohort

As outlined in the Materials and Methods section, we established a study cohort consisting of 12 PTC cases with the hot spot mutations (C228T and C250T), 8 cases with the C216T mutation, and 120 cases with the wildtype genotype (Fig. 1). In cases with hot spot mutations (C228T and C250T), the median age was 58 years (range 37–77), and the female-to-male ratio was 1.4. The median tumor diameter was 2.1 cm, with a range of 0.7–5.0 cm. The number of cases categorized as low, intermediate, and high ATA recurrence risk were 0, 7, and 5, respectively. The number of cases categorized as AJCC staging I, II, III, and IV were 5, 7, 0, and 0, respectively (Table 1).

**Table 1:** Clinicopathologic characteristics of papillary thyroid carcinoma patients with TERT promoter mutation

Case No.	TERT promoter mutation	Age (yr)	Sex	Tumor size (cm)	Histologic variant	Multifocality	Vascular invasion	Extrathyroidal extension	Hashimoto’s thyroiditis	ATA recurrence risk	AJCC staging				BRAF V600E mutation
											pT	pN	M	Stage	
1	C228T	58	Male	1.5	Classic	*N*	*N*	None	*N*	Intermediate	1	1	0	2	Present
2	C228T	58	Female	1.4	Classic	*N*	*N*	Minimal	*N*	Intermediate	1	1	0	1	Present
3	C228T	77	Male	2.5	Classic	Y	Y	Minimal	*N*	High	2	1	0	2	Present
4	C228T	63	Female	2.4	Tall cell	*N*	*N*	Minimal	*N*	Intermediate	2	0	0	1	None
5	C228T	67	Female	2.1	Classic	Y	*N*	Minimal	*N*	Intermediate	2	1	0	2	Present
6	C228T	64	Female	0.9	Tall cell	Y	*N*	Minimal	*N*	Intermediate	1	1	0	2	Present
7	C228T	63	Female	5.0	Classic	*N*	*N*	Gross	*N*	High	3	1	0	2	Present
8	C228T	65	Female	2.4	Classic	*N*	Y	Minimal	*N*	High	2	1	0	2	Present
9	C228T	56	Female	4.2	Tall cell	*N*	*N*	Gross	*N*	High	3	1	0	2	Present
10	C228T	37	Male	0.7	Classic	Y	*N*	None	*N*	Intermediate	1	1	0	1	Present
11	C250T	51	Male	2.3	Classic	Y	*N*	Minimal	*N*	High	2	1	0	1	Present
12	C228T	52	Male	1.7	Classic	*N*	*N*	Minimal	*N*	Intermediate	1	1	0	1	None
13	C216T	30	Male	0.6	Classic	*N*	*N*	None	*N*	Low	1	0	0	1	Present
14	C216T	50	Female	0.6	Classic	*N*	*N*	None	*N*	Low	1	1	0	1	Present
15	C216T	37	Female	0.3	Classic	*N*	*N*	None	*N*	Low	1	0	0	1	Present
16^#^	C216T	69	Female	0.6	Classic	NA	*N*	None	NA	NA	1	NA	NA	NA	Present
17^*^	C216T	44	Female	0.4	Classic	*N*	NA	None	NA	Low	1	0	0	1	Present
18^*^	C216T	55	Female	0.5	Classic	*N*	NA	None	NA	Low	1	0	0	1	Present
19^*^	C216T	29	Male	1.0	Classic	Y	NA	Minimal	NA	Intermediate	1	1	0	1	Present
20^*^	C216T	54	Male	1.2	Classic	*N*	NA	Minimal	NA	Intermediate	1	0	0	1	Present

Y, yes; N, no; NA, not available; # enrolled from the reference 15; * enrolled from the reference 16

For cases with the C216T mutation, the median age was 44 years (range 29–69) and the female-to-male ratio was 1.7. The median tumor diameter was 0.6 cm, with a range of 0.3–1.2 cm. The number of cases categorized as low, intermediate, and high ATA risk were 5, 2, and 0, respectively. The number of cases categorized as AJCC staging I, II, III, and IV were 7, 0, 0, and 0, respectively (Table 1).

For cases with the wildtype genotype, the median age was 38 years (range 21–73) and the female-to-male ratio was 3.4. The median tumor diameter was 0.8 cm, with a range of 0.1–2.6 cm. The number of cases categorized as low, intermediate, and high ATA risk were 65, 48, and 7, respectively. The number of cases categorized as AJCC staging I, II, III, and IV were 111, 9, 0, and 0, respectively (Table 2).

**Table 2:** Baseline characteristics of papillary thyroid carcinoma patients with wildtype genotype of TERT promoter

Characteristic	No. (%) (Total = 120)
**Age at diagnosis (yr)**	
< 55	104 (86.7%)
≥ 55	16 (13.3%)
Median(range)	38 (21–73)
**Sex**	
Female	93 (77.5%)
Male	27 (22.5%)
**Histologic variant**	
Classic	100 (83.3%)
Follicular	7 (5.8%)
Tall cell	5 (4.2%)
Diffuse sclerosing	5 (4.2%)
Solid	3 (2.5%)
**Multifocality**	
Yes	37 (30.8%)
No	83 (69.2%)
**Vascular invasion**	
Yes	7 (5.8%)
No	113 (94.2%)
**Extrathyroidal extension**	
Absent	82 (68.3%)
Minimal	36 (30%)
Gross (T3b)	2 (1.7%)
Gross (T4)	0 (0%)
**Hashimoto’s thyroiditis**	
Yes	24 (20%)
No	96 (80%)
**Tumor size (cm)**	
≤ 1	85 (70.8%)
> 1, ≤ 2	32 (26.7%)
> 2, ≤ 4	3 (2.5%)
> 4	0 (%)
**Pathologic T category**	
T1	116 (96.7%)
T2	3 (2.5%)
T3	1 (0.8%)
T4	0 (%)
**Pathologic N category**	
N0	59 (49.2%)
N1	61 (50.8%)
**ATA recurrence risk**	
Low	65 (54.2%)
Intermediate	48 (40%)
High	7 (5.8%)
**AJCC staging**	
I	111 (92%)
II	9 (8%)
III	0 (0%)
IV	0 (0%)
**BRAF V600E mutation**	
Present	104 (86.7%)
None	16 (13.3%)

### The influence of C216T and hot spot mutations on the clinicopathologic characteristics of PTC cases

In comparison with the wildtype genotype, the C216T mutation did not impose remarkable influences on the clinicopathologic profiles of PTCs. There was no significant difference in variables such as age, histologic variant, extrathyroidal invasion, ATA recurrence risk, AJCC staging, and BRAF mutation between the two groups (Table 3).

**Table 3:** Correlation between TERT promoter mutation and clinicopathologic characteristics in patients with papillary thyroid carcinoma

Variables	TERT promoter mutation					*p* Value		
	Wildtype (A)	C228T, C250T (B)	C216T (C)	Total		A vs. B	A vs. C	B vs. C
**Age (yr)**						< 0.001	0.313	0.065
<55	104 (92.0%)	3 (2.7%)	6 (5.3%)	113				
≥ 55	16 (59.3%)	9 (33.3%)	2 (7.4%)	27				
Total	120	12	8	140				
Mean ± sd	40.28 ± 10.78	59.25 ± 9.95	46.00 ± 13.73			< 0.001	0.155	0.022
**Sex**						0.91	0.389	1.000
Female	93 (88.6%)	7 (6.7%)	5 (4.8%)	105				
Male	27 (77.1%)	5 (14.3%)	3 (8.6%)	35				
Total	120	12	8	140				
**Histologic variant**						0.063	1.000	0.242
Non-aggressive	112 (86.8%)	9 (7%)	8 (6.2%)	129				
Aggressive	8 (72.7%)	3 (27.3%)	0 (0%)	11				
Total	120	12	8	140				
**Multifocality**						0.519	0.673	0.333
Yes	37 (86%)	5 (11.6%)	1 (2.3%)	43				
No	83 (86.5%)	7 (7.3%)	6 (6.3%)	96				
Total	120	12	7	139				
**Vascular invasion**						0.190	1.000	1.000
Yes	7 (77.8%)	2 (22.2%)	0 (0%)	9				
No	113 (89%)	10 (7.9%)	4 (3.1%)	127				
Total	120	12	4	136				
**Extrathyroidal extension**						0.001	1.000	0.019
Yes	38 (76%)	10 (20%)	2 (4%)	50				
No	82 (91.1%)	2 (2.2%)	6 (6.7%)	90				
Total	120	12	8	140				
**Hashimoto’s thyroiditis**						0.122	1.000	NA
Yes	24 (100%)	0 (0%)	0 (0%)	24				
No	96 (86.5%)	12 (10.8%)	3 (2.7%)	111				
Total	120	12	3	135				
**Tumor size (cm)**						0.003	0.136	0.002
Mean ± sd	0.88 ± 0.43	2.26 ± 1.25	0.65 ± 0.30					
**Pathologic T category**						< 0.001^#^	1.000^#^	0.015^#^
T1	116 (89.9%)	5 (3.9%)	8 (6.2%)	129				
T2	3 (37.5%)	5 (62.5%)	0 (0%)	8				
T3	1 (33.3%)	2 (66.7%)	0 (0%)	3				
Total	120	12	8	140				
**Pathologic N category**						0.031	0.440	0.045
N0	59 (89.4%)	2 (3%)	5 (7.6%)	66				
N1	61 (83.6%)	10 (13.7%)	2 (2.7%)	73				
Total	120	12	7	139				
**Metastatic nodal number**	1.83 ± 2.73	6.75 ± 5.12	0.33 ± 0.82			0.007	0.183	0.001
**ATA recurrence risk**						< 0.001^*^	0.458^*^	0.002^*^
Low	65 (92.9%)	0 (0%)	5 (7.1%)	70				
Intermediate	48 (84.2%)	7 (12.3%)	2 (3.5%)	57				
High	7 (58.3%)	5 (41.7%)	0 (0%)	12				
Total	120	12	7	139				
**AJCC staging**						< 0.001	1.000	0.017
I	111(90.2%)	5 (4.1%)	7 (5.7%)	123				
II	9 (56.3%)	7 (43.8%)	0 (0%)	16				
Total	120	12	7	139				
**BRAF V600E mutation**						0.668	0.495	0.595
Present	104 (85.2%)	10 (8.2%)	8 (6.6%)	122				
None	16 (88.9%)	2 (11.1%)	0 (0%)	18				
Total	120	12	8	140				

NA, not available; # The comparison was performed between the T1 group and the T2/T3 group; * The comparison was performed between the low risk group and the intermediate/high risk group

In contrast, the hot spot mutations were found to be significantly associated with older age (p < 0.001, p = 0.022) and larger tumor size (p = 0.003, p = 0.002) when compared with the wildtype genotype and C216T mutation, respectively. Additionally, there was a higher incidence of extrathyroidal extension (*p* = 0.001, *p* = 0.019) in cases with hot spot mutations. Furthermore, the hot spot mutations exhibited significant correlations with higher T category (*p* < 0.001, *p* = 0.015), increased nodal metastasis (*p* = 0.031,  *p*= 0.045), a greater number of metastatic lymph nodes (*p* = 0.007, *p*= 0.001), and elevated ATA risk (*p* < 0.001, *p* = 0.002) and AJCC staging (*p* < 0.001, *p* = 0.017) (Table 3).

### The expression status of S100A10 in PTCs with and without TERTp mutations

**Fig. 2:**
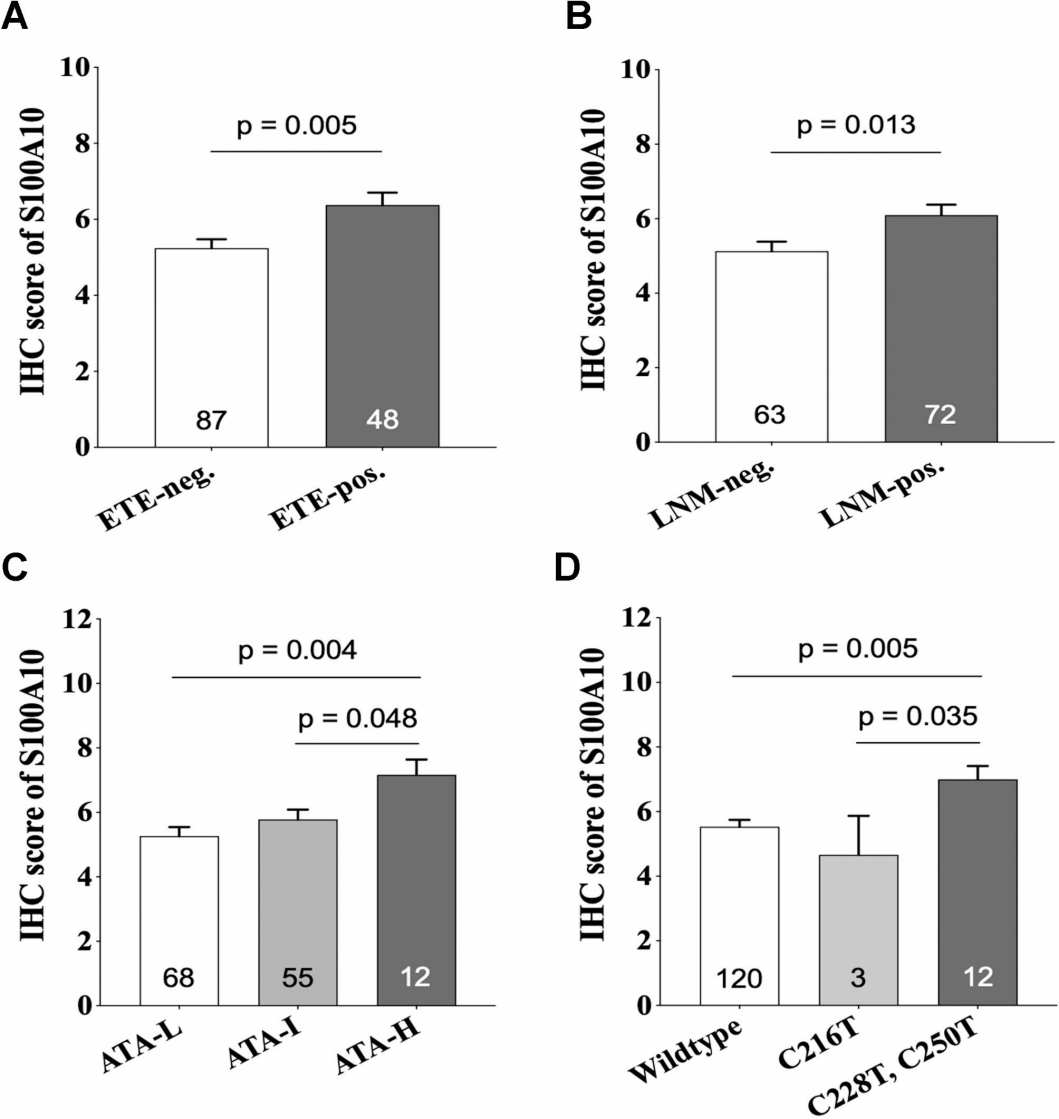
The correlations between the expression levels of S100A10 protein and the phenotype-genotype characteristics of papillary thyroid carcinoma (PTC). The increased expression of S100A10 protein was positively associated with extrathyroidal extension (**A**), lymph node metastasis (**B**), and ATA recurrence risk (**C**) in PTC patients. In contrast to cases with the wildtype genotype, the expression levels of S100A10 protein were significantly up-regulated in PTC cases with the hot spot mutations (C228T and C250T), while there was no significant change in cases with the C216T mutation (**D**). ETE, extrathyroidal extension; LNM, lymph node metastasis; ATA-L, ATA recurrence risk-low; ATA-I, ATA recurrence risk-intermediate; ATA-H, ATA recurrence risk-high

**Fig. 3:**
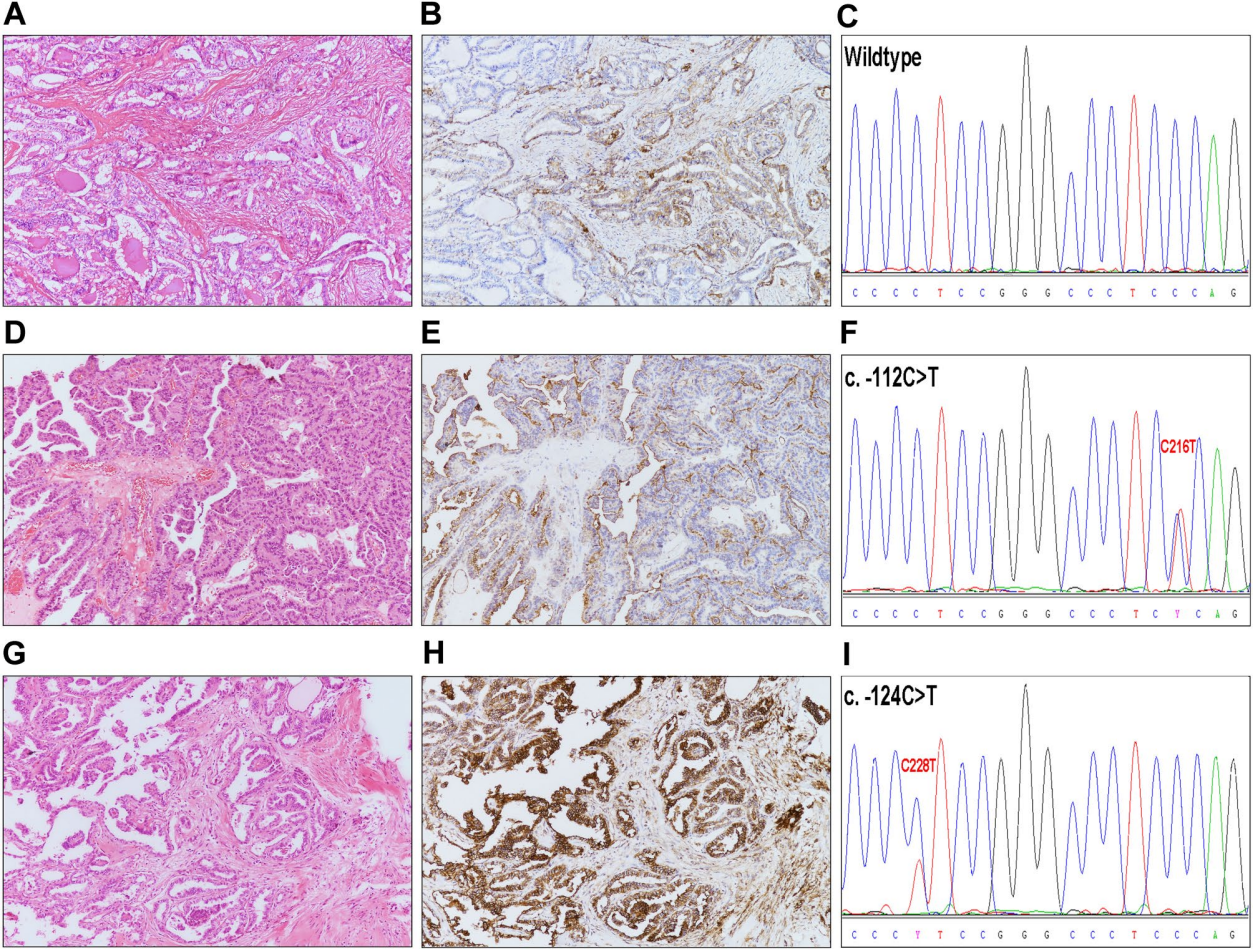
The immunostaining characteristics of S100A10 protein in cases of papillary thyroid carcinoma (PTC) with and without TERTp mutation. (**A-C**) showed a representative case of PTC with the wildtype genotype. The tumor’s morphology was displayed in figure **A** (H&E, 200×). The tumor cells showed a weak immunostaining with S100A10 (**B**, 200×). No TERTp mutations were identified in this case. The DNA sequence encompassing the c.-124 and c.-112 sites was visualized in figure **C**. (**D-F**) demonstrated a representative case with the C216T mutation. The tumor’s morphology was displayed in figure **D** (H&E, 200×). The tumor cells showed a weak immunostaining with S100A10 (**E**, 200×). The C216T mutation, located at position c.-112, was detected in this case, and a *de novo* TCTC motif was generated (**F**). (**G-I**) presented a representative case with the C228T mutation. The tumor’s morphology was displayed in figure **G** (H&E, 200×). The tumor cells exhibited strong immunostaining with S100A10 (**H**, 200×). The C228T mutation, located at position c.-124, was detected in this case, and a *de novo* CCTT motif was generated (**I**)

We first evaluated the clinical significance of S100A10 protein expression in the cohort of PTC cases from our institution, which included the 120 patients with wildtype genotype, 12 patients with hotspot mutations, and 3 patients with C216T mutations (Supplementary Table). The results showed a positive correlation between the expression of S100A10 protein and extrathyroidal extension (*p* = 0.005) (Fig. 2A), lymph node metastasis (*p* = 0.013) (Fig. 2B), and upgraded ATA recurrence risk (*p* = 0.023) (Fig. 2C). The subsequent analysis showed that, in comparison to the wildtype cases of PTC, the expression of S100A10 was significantly up-regulated in cases with the hot spot mutations (*p* = 0.030) (Fig. 2D), while it was not markedly changed in cases with the C216T mutation (Fig. 3).

Based on the aforementioned findings, we conducted further analysis of the S100A10 mRNA expression characteristics in PTC cases from the TCGA database. Consistent with the immunostaining findings, the expression of S100A10 mRNA was found to be significantly correlated with extrathyroidal extension (*p* < 0.001) (Supplementary Fig. 2A) and lymph node metastasis (*p* < 0.001) (Supplementary Fig. 2B). Moreover, the mRNA levels of S100A10 were significantly higher in cases with the hot spot mutations compared to those with the wildtype genotype (*p* = 0.011) (Supplementary Fig. 2C).

## Discussion

The C228T and C250T are well known hot spot mutations in TERTp. As demonstrated in several previous studies, the two mutations are mutually exclusive, with C228T being more prevalent than C250T [35, 36]. In contrast, the C216T mutation, which is in close proximity to the two hot spot mutations in nucleotide position, was initially documented in PTCs in 2020, and only 5 cases have been reported to date. According to two recent studies discussing this mutation, the frequency of the C216T mutation was lower than that of C228T, but higher than that of C250T [15, 16]. In one of those study, the mutant frequencies of C228T, C216T, and C250T were 1.9%, 0.6%, and 0.3%, respectively. In another one, these frequencies were 1.9%, 0.16%, and 0%, respectively. Our current findings were consistent with their data, showing that the frequency of C216T (0.19%) fell between that of C228T (0.68%) and C250T (0.06%). The findings indicated that, although the C216T mutation was rarely reported, its frequency was at least higher than that of the C250T mutation in PTCs. Furthermore, based on our current study and the two earlier investigations mentioned above [15, 16], the C216T mutation was found to be mutually exclusive with the C228T and C250T mutations. Additionally, our study demonstrated that, similar to C228T and C250T, the C216T mutation was somatic in nature.

The primary motivation we initiated this study was to address the inquiries from clinicians and affected patients regarding the clinical significance of the C216T mutation, which had not yet been fully understood. The presented findings indicated that the C216T mutation did not lead to an increased risk of recurrence and mortality in PTC patients. This was supported by the lack of significant differences in the ATA recurrence stratification and AJCC staging between cases with this mutation and those with the wildtype genotype. The findings were largely in consistent with the results from the study of Kim et al. [16]. It meant that the C216T did not appear to be a detrimental mutation and unlikely brought an unfavorable consequence. Conversely, the hot spot mutations were unequivocally associated with higher risks of recurrence and mortality, as also supported by several previous studies [11–14].

It has been suggested that the hot spot mutations of TERT generate a *de novo* consensus binding site (CCTT) for transcription factor E-twenty-six (ETS). The subsequent binding reactions enhance the transcription of TERT, ultimately leading to telomere maintenance and the sustained proliferation of cancer cells [37, 38]. In contrast to the hot spot mutations, the C216T mutation formed a TCTC motif instead of CCTT (Fig. 3). Based on the aforementioned scenario, the C216T mutation lacked the structural basis for the activation of the ETS-induced downstream effects.

In addition to potentially restoring telomere integrity as stated above, the hot spot mutations of TERTp have been found to induce abnormal expression of a series of genes, which may concurrently contribute to the pathogenesis of PTC. In an ongoing investigation, we conducted a screening for differentially expressed proteins in PTCs with the hot spot mutations using the method of mass spectrometry. S100A10 was identified as one of the significantly up-regulated proteins (data not shown). Our present investigation revealed a significant increase in S100A10 expression in cases with hot spot mutations, as opposed to those with the wildtype genotype. However, there was no remarkable change in expression in cases with the mutant C216T. S100A10 has been shown to play a crucial role in promoting invasion and metastasis in several types of cancer by stimulating plasmin-mediated degradation of the extracellular matrix. Previous research has demonstrated that elevated expression of S100A10 was closely linked to the lymph node metastasis of PTC [39]. Our present findings provided further evidence of the pro-cancer role of S100A10 in PTC, as indicated by its correlation with extrathyroidal extension, nodal metastasis, and the risk of recurrence. Thus, S100A10 might partially mediate and/or synergistically augment the pro-invasive effect of the hot spot mutations on PTCs. The current study did not focus on exploring the mechanism that connects the hot spot mutations and S100A10 overexpression. Noteworthily, the TERT protein has been shown to increase the transcription of IL-1β and basic fibroblast growth factor (bFGF) [40], while IL-1β and bFGF are known to be strong inducers for S100A10 syntheses [41]. We speculate that these molecular events may serve as the potential basis for the cross-talk between the hot spot mutations and increased S100A10 expression, which deserves further investigation.

The main limitation of the present study was the small sample size of enrolled cases with the C216T mutation, although it was the largest case cohort known to us so far. However, there were still detectable differences in clinicopathologic characteristics between C216T and hot spot mutations, as demonstrated by our current study and Kim et al.‘s investigation [16]. More importantly, the structural disparity in the *de novo* nucleotide motif resulting from the C216T and hot spot mutations, as well as the variation in the expression level of S100A10 between them, may account for their distinct clinical outcomes. The low expression of S100A10 in the C216T mutant cases is also expected to to be validated with a larger sample size. Nevertheless, the strong correlation between the increased expression of S100A10 and the hot spot mutations offers new insight into understanding the pro-invasive role of the hot spot mutations in PTC.

## Summary

The present study demonstrated that the C216T mutation was somatic and showed mutual exclusivity with the hot spot mutations. The mutation frequency of C216T was between that of C228T and C250T. Furthermore, the C216T mutation did not appear to have pathogenic effects on the aggressiveness of PTC. The findings suggested that post-surgery treatments and surveillance strategies used for patients with hot spot mutations may not be necessary for patients with C216T mutation. Moreover, we have demonstrated for the first time that the hot spot mutations of TERTp are closely associated with the upregulated expression of S100A10. The latter may partially mediate and/or coordinately enhance the pro-invasive function of the hot spot mutations on PTCs. Additional research is expected to further confirm the role of the C216T mutation with a larger sample size and to elucidate the underlying mechanism connecting the increased expression of S100A10 and the hot spot mutations in PTC.

## Electronic supplementary material

Below is the link to the electronic supplementary material.

Supplementary Material 1

Supplementary Material 2

## Data Availability

Data is provided within the manuscript or supplementary information files.
