# Supplementary material for: The impact of C216T and hot spot mutations of the TERT promoter on the clinicopathologic characteristics and S100A10 expression in papillary thyroid carcinoma: a comparative study
Source: Diagn Pathol. 2025 Feb 11;20:15. doi: 10.1186/s13000-025-01613-6 (PMC11816782; doi:10.1186/s13000-025-01613-6)
Supplement: Supplementary file 1 — Supplementary Material 1 [file 13000_2025_1613_MOESM1_ESM.docx]

**
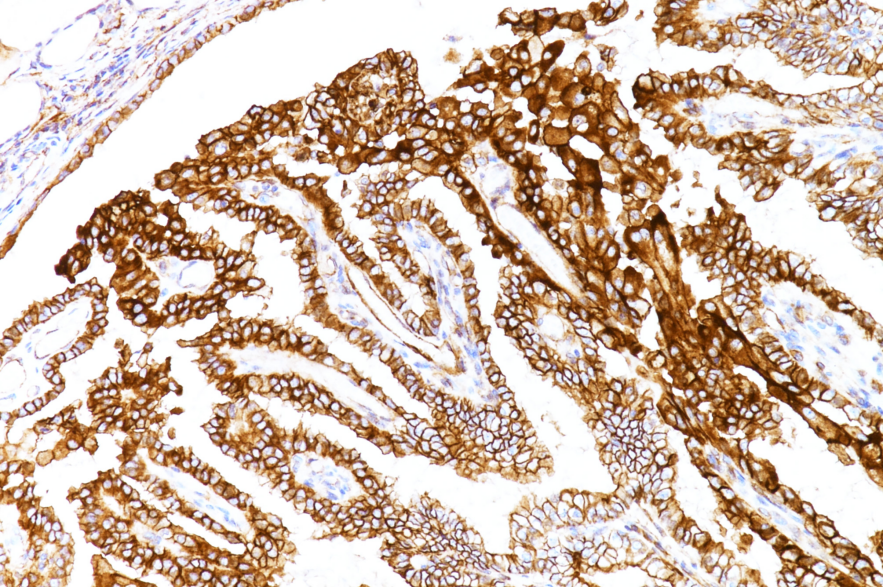
**

**Supplementary Fig. 1** Representative strong immunostaining of S100A10 protein in papillary thyroid carcinoma. It was set as the benchmark for strong S100A10 staining intensity (×200).


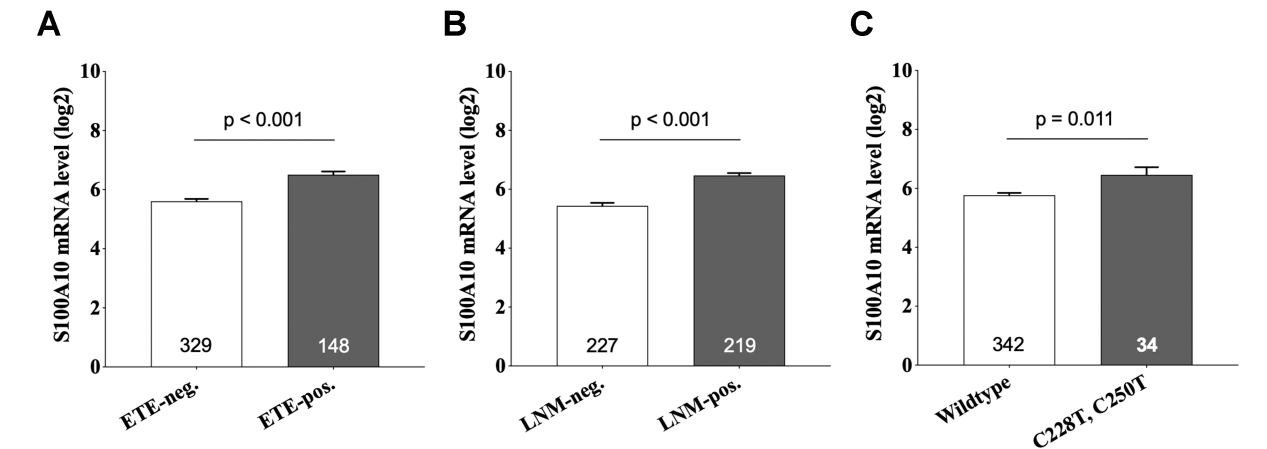


**Supplementary Fig. 2** The correlation between the expression levels of S100A10 mRNA and the phenotype-genotype characteristics of papillary thyroid carcinoma (PTC). Increased expression of S100A10 mRNA was positively associated with extrathyroidal extension (**A**) and lymph node metastasis (**B**) in PTC. The expression levels of S100A10 mRNA were significantly higher in cases of PTC with the hot spot mutations (C228T and C250T) compared to those with the wildtype genotype of TERTp.
