# Supplementary material for: The impact of C216T and hot spot mutations of the TERT promoter on the clinicopathologic characteristics and S100A10 expression in papillary thyroid carcinoma: a comparative study
Source: Diagn Pathol. 2025 Feb 11;20:15. doi: 10.1186/s13000-025-01613-6 (PMC11816782; doi:10.1186/s13000-025-01613-6)
Supplement: Supplementary file 2 — Supplementary Material 2 [file 13000_2025_1613_MOESM2_ESM.docx]

**Supplementary Table** Correlation between the expression of S100A10 protein and clinicopathologic characteristics in patients with papillary thyroid carcinoma

| **Variables** | n (Total =135) | **S100A10 (mean ± sd)** | p Value |
| --- | --- | --- | --- |
|  |  |  |  |
| **Age** |  |  | 0.838 |
| < 55 | 110 | 5.66 ± 2.30 |  |
| ≥ 55 | 25 | 5.56 ± 2.22 |  |
| **Sex** |  |  | 0.981 |
| Female | 102 | 5.64 ± 2.11 |  |
| Male | 33 | 5.64 ± 2.12 |  |
| **Histologic variant** |  |  | 0.793 |
| Non-aggressive | 124 | 5.63 ± 2.32 |  |
| Aggressive | 11 | 5.81 ± 1.83 |  |
| **Multifocality** |  |  | 0.623 |
| Yes | 42 | 5.50 ± 2.27 |  |
| No | 93 | 5.71 ± 2.30 |  |
| **Vascular invasion** |  |  | 0.964 |
| Yes | 9 | 5.67 ± 1.41 |  |
| No | 126 | 5.64 ± 2.33 |  |
| **Extrathyroidal extension** |  |  | 0.005 |
| Yes | 48 | 6.38 ± 2.29 |  |
| No | 87 | 5.24 ± 2.18 |  |
| **Hashimoto's thyroiditis** |  |  | 0.103 |
| Yes | 24 | 6.33 ± 2.31 |  |
| No | 111 | 5.50 ± 2.26 |  |
| **Pathologic T category** |  |  | 0.055 |
| T1 | 124 | 5.53 ± 2.30 |  |
| T2--3 | 11 | 6.91 ± 1.64 |  |
| **Pathologic N category** |  |  | 0.013 |
| N0 | 63 | 5.13 ± 2.05 |  |
| N1 | 72 | 6.10 ± 2.39 |  |
| **ATA recurrence risk** |  |  | 0.023 |
| Low | 68 | 5.26 ± 2.30 |  |
| Intermediate | 55 | 5.78 ± 2.25 |  |
| High | 12 | 7.17 ± 1.64 |  |
| **AJCC staging** |  |  | 0.668 |
| Ⅰ | 119 | 5.61 ± 2.32 |  |
| Ⅱ | 16 | 5.88 ± 2.00 |  |
| **TERT mutation** |  |  | 0.030 |
| Wildtype | 120 | 5.53 ± 2.32 |  |
| C216T | 3 | 4.67 ± 2.08 |  |
| C228T, C250T | 12 | 7.00 ± 1.41 |  |
| **BRAF V600E mutation** |  |  | 0.413 |
| Present | 117 | 5.58 ± 2.26 |  |
| None | 18 | 6.06 ± 2.41 |  |
